# Validity of the Loewenstein–Acevedo Scales of Semantic Interference and Learning (LASSI-L) for Mexican Subjects with Mild and Moderate Cognitive Impairments

**DOI:** 10.3390/geriatrics10060141

**Published:** 2025-10-30

**Authors:** A. Kammar-García, P. Peña-Gonzalez, J. Sigg-Alonso, T. Álvarez-Cisneros, P. Roa-Rojas

**Affiliations:** 1 Instituto Nacional de Geriatría, Ciudad de México 10200, Mexico; akammar@inger.gob.mx (A.K.-G.); paola.penago@gmail.com (P.P.-G.); talvarez@inger.gob.mx (T.Á.-C.); 2 Laboratory of Psychophysiology, Department of Behavioral and Cognitive Neurobiology, Instituto de Neurobiología, Universidad Nacional Autónoma de México (UNAM), Campus Juriquilla, Querétaro 76230, Mexico; jorgesigg@gmail.com

**Keywords:** cognitive decline, Alzheimer disease, memory, neuropsychological assessment

## Abstract

**Background/Objectives:** Alzheimer’s disease (AD) often begins with episodic memory deficits, detectable in Mild Cognitive Impairment (MCI). The Loewenstein–Acevedo Scales of Semantic Interference and Learning (LASSI-L) shows promise for early detection, but lacks validation in Mexico. **Methods**: We assessed 355 adults ≥ 60 years, classified as cognitively healthy (CHG), MCI, or mild AD, using DSM-V criteria. Participants completed neuropsychological testing including the LASSI-L. Construct, concurrent, and predictive validity were analyzed via ANOVA, correlations with the Hopkins Verbal Learning Test (HVLT), and logistic regression models controlling for age, education, and comorbidity. **Results**: LASSI-L scores significantly differed between groups (*p* < 0.0001), with recovery from proactive interference best discriminating CHG from MCI and mild AD. Strong correlations with HVLT indices supported concurrent validity. Predictive models identified semantically cued recall and free recall (CRA2 and FRB1) as robust markers, independent of education. **Conclusions**: LASSI-L is a valid, accessible tool for identifying typical AD-related memory impairment in older Mexican adults, supporting earlier diagnosis in low-biomarker-access settings.

## 1. Introduction

With the aging of the global population, the prevalence of neurocognitive disorders such as Alzheimer’s disease (AD) is steadily increasing [1]. This condition poses a significant challenge to healthcare systems, as it ultimately results in complete dependence of the affected individual. Consequently, early [2] and timely diagnosis is of paramount importance. A four-phase diagnostic process is currently recommended. In Phase 0, a basic assessment is performed to establish a probable diagnosis of Mild Cognitive Impairment (MCI) or dementia. In Phase 1, a specific diagnostic hypothesis is formed based on the classification of deficits into a clinical phenotype. At this point, a comprehensive neuropsychological evaluation and cognitive profile analysis are advised. In phases 2 and 3, biomarker analysis is recommended [3]. Throughout this process, a combined clinical–biological approach should be maintained, in which the presence of a specific clinical phenotype is linked to specific biomarkers [4].

MCI refers to cognitive impairment that has a detectable functional impact on complex daily activities but does not result in loss of independence [5,6]. In Mexico, its prevalence is estimated at 18% [7]. MCI is a known factorial risk to dementia in general [8] and to AD in up to 23.8% of cases [9]. The clinical phenotype of MCI most often associated with AD is the hippocampal-type amnestic syndrome [4], characterized by episodic memory deficits. Evidence suggests prevalence can go from 45.9% to 64% depending on measuring techniques [10,11]. Individuals with this syndrome perform worse than healthy controls on free and delayed recall tasks [12,13], with no improvement when semantic cues are provided. Other cognitive domains may also be affected.

Several instruments assess episodic memory by modifying the traditional serial word-learning paradigm to identify this phenotype [14]. One such tool is the Free and Cued Selective Reminding Test (FCSRT) [15,16], which has proven sensitive in detecting prodromal AD-related memory impairments [17]. It produces indices of immediate and delayed recall and evaluates the effect of semantic cues [18]. Some instruments have demonstrated even greater sensitivity [19], notably the Loewenstein–Acevedo Scales of Semantic Interference and Learning (LASSI-L) [20].

The LASSI-L involves learning two lists of 15 words each, clustered into three semantic categories, using active encoding by deliberately engaging participants with the material through reading and listening, maximizing the depth of processing of words to be remembered. It assesses delayed recall with and without cues, retroactive interference (RI), proactive interference (PI), and PI recovery [14]. Its biological validity has been demonstrated through evidence indicating an association between specific scores (PI and PI recovery) and biomarker positivity, such as elevated Tau levels in cerebrospinal fluid and atrophy of brain regions associated with Alzheimer’s disease [21,22]. Also, it has demonstrated the ability to identify individuals at high risk of converting from MCI to AD [23] and to detect alterations in cognitively normal individuals [24,25], those with subjective memory complaints [26], MCI [27], and mild AD [28]. Originally developed in a verbal format, the LASSI-L requires no more than 30 min to administer, does not need specialist interpretation, and is accessible to individuals with varying educational levels. A brief version and a computerized version are also available [29,30].

In summary, evidence indicates that the clinical phenotype associated with early and mild AD is characterized by episodic memory deficits detectable with tests such as the LASSI-L. These instruments are particularly useful for identifying deficits during the pre-dementia stage (MCI). However, most have not been validated in Latin America or Mexico. The present study seeks to validate the LASSI-L in Mexican participants by assessing construct, concurrent, and predictive validity in individuals with MCI and mild AD. This is particularly relevant in Latin America, where biomarker-based diagnosis is not widely available, and such tools could enable earlier and more accurate detection.

## 2. Materials and Methods

### 2.1. Subjects

A total of 355 individuals aged over 60 years were included in the present analysis. Participants were recruited through the National Institute of Geriatrics (INGER) at various day centers. Based on DSM-V diagnostic criteria for Minor Neurocognitive Disorder (mND) and Major Neurocognitive Disorder (MND), along with expert clinical judgment, participants were classified into three groups: cognitively healthy group (CHG), group with MCI or Minor Neurocognitive Disorder (mND), and group with mild AD or Major Neurocognitive Disorder (MND).

Inclusion criteria for the CHG were: no self-reported or informant-reported concerns regarding cognitive decline, ability to independently perform complex activities of daily living (Lawton score = 8, Barthel score = 100) [31,32], a MoCA score of 26 [33,34] or higher and other cognitive measures with less than 1.0 SD below normal limits for age and education (Category fluency and Trails A and B) [35,36]. Inclusion criteria for the MCI group were: concern from the participant, an informed third party, or a caregiver regarding a mild decline in cognitive abilities; a MoCA score below 26 and evidence of mild impairment in one or more cognitive domains reflected by neuropsychological test performance one to two standard deviations below age and education normative data (Category fluency and Trails A and B); and preserved ability to independently perform complex activities of daily living (Lawton score = 7–8, Barthel score = 100). Inclusion criteria for the mild AD group were: a MoCA score below 24, evidence of moderate impairment in one or more cognitive domains, indicated by neuropsychological test performance at least two standard deviations below age normative data (Category fluency and Trails A and B); and evidence of cognitive deficits interfering with the ability to independently perform activities of daily living (Lawton score < 7, Barthel score ≤ 95). Neuropsychological and other tests are described in the procedure.

### 2.2. Procedure

An invitation to participate was sent via INGER to various day centers. All participants who verbally agreed to participate completed the informed consent process and signed the corresponding form. Each participant underwent a battery of neuropsychological tests, health questionnaires, and functional assessment scales. Evaluations were conducted in two private sessions, each lasting approximately one hour, scheduled one week apart. Assessments were performed individually, although caregivers could be present upon request by the participant or themselves. All instruments were administered by a trained mental health professional.

During the first session, all tests were administered as follows: health history and sociodemographic questionnaire, detailed clinical history, MoCA [33,34], depression scales (CESD-7 and Geriatric Depression Scale) [37,38], and the Hopkins Verbal Learning Test [39]. The second session included the Charlson Comorbidity Index, Lawton & Brody functioning scale and Barthel’s dependency scale [31,32], the LASSI-L, verbal fluency tasks [40], and the Trail Making Test [41]. In the AD group, a comprehensive review of clinical history and medical records was undertaken to exclude alternative causes of cognitive impairment. In all cases, neuroimaging studies (magnetic resonance imaging or computed tomography) were available, confirming the absence of recent vascular lesions (within six months prior to the study). Also, to ensure mild severity, the Clinical Dementia Rating (CDR) scale was also applied (Global CDR scale of 1.0) [42] Upon completion, group classifications (CHG, MCI, or mild AD) were reviewed and confirmed by an expert.

### 2.3. Loewenstein–Acevedo Scales of Semantic Interference and Learning

**Figure 1:**
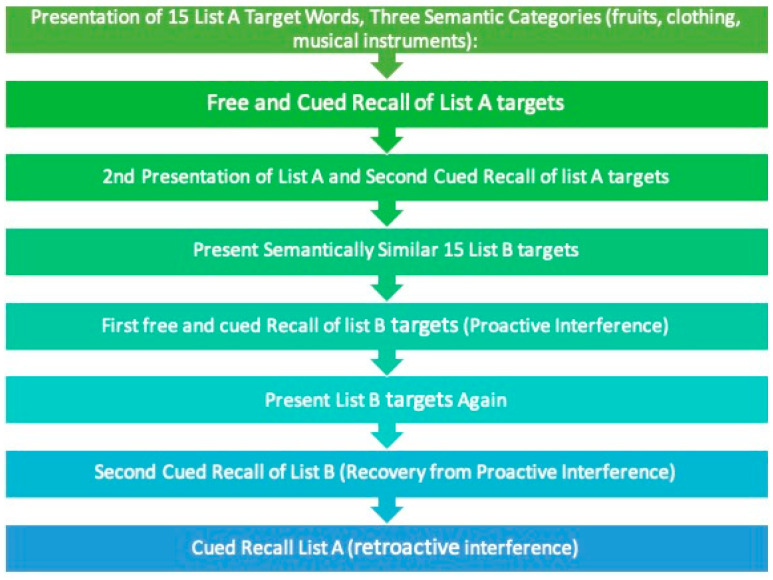
Graphic description of LASSI-L. Based on Loewenstein et al. [14].

For this study, the original Spanish version of the LASSI-L was used. For this purpose, some words were changed as follows: From list A “Armónica”, “banana”, “chaqueta”, and “media” were replaced with “tambor”, “plátano”, “chamarra”, and “calcetines”. From list B “melocotón”, “clarinete”, and “acordeón” were replaced with “durazno”, “pandero”, and “maraca”. No changes were made to semantic categories and order of presentation. LASSI-L procedure is as follows: the examiner presents a list of 15 common words (List A) with intermixed semantic categories (fruits, musical instruments, and clothing). The participant reads the list of words aloud at four-second intervals between each word. After reading, the participant recalls the words (free recall, FRA1), followed immediately by semantically cued recall (CRA1). The same procedure is repeated for a second cued recall (CRA2). A semantically related list of 15 words (List B) is then presented and learned in the same manner, followed by free recall (FRB1) and semantically cued recall (CRB1), then repeated for a second cued recall (CRB2). This is followed by delayed free recall of List A (SdFRA) and delayed cued recall of List A (SdCRA). Finally, delayed total free recall of all words from both lists is performed 20 min later (DR). For a graphic description see Figure 1.

For scoring, the examiner records the number of correct words from both target lists and the number of intrusions (words not included on the lists) on each attempt. Each attempt yields a score between 0 and 15 for both correct words and intrusions. Nine measures are obtained: Free recall List A (FRA1), Free recall List B (FRB1), Semantically cued recall List A1 (CRA1), Semantically cued recall List A2 (CRA2), Semantically cued recall List B1 (CRB1), Semantically cued recall List B2 (CRB2), Free deferred recall (SdFR), semantically cued deferred recall (SdCRA) and delayed total recall (DR).

### 2.4. Statistical Analysis

A descriptive analysis of the total sample was conducted, stratified by group, to define distribution similar to normal we use the asymmetry (±0.5) and kurtosis stats (±2.0). Discriminant validity was obtained examining whether the LASSI-L could differentiate between groups. A One-way ANOVA was conducted to compare test performance between groups (CHG, MCI, AD). Tukey’s post hoc tests were used to identify group differences, the effect size of cognitive status on each evaluated variable was calculated using partial eta squared (η^2^p). ANOVA assumptions were assessed through residual analysis (normality [by QQ-plots, asymmetry and kurtosis stats] and independence of residuals [Durbin–Watson test]) and Levene’s test for homogeneity of variances.

Criterion validity was assessed via concurrent validity by comparing LASSI-L performance with that of the Hopkins Verbal Learning Test (HVLT). Spearman correlations were calculated between LASSI-L indices (FRA1, CRA1, CRA2, FRB1, CRB1, CRB2, SdFRA, SdCRA, DR) and HVLT indices (immediate recall free one (IFR1), immediate recall interference one (IRI1), delayed recall free one (DLR1), delayed recall with cues one (DLRC1), delayed recall free two (DLR2), and delayed recall with cues two (DLRC2)).

Predictive validity was examined through binary logistic regression followed by ordinal logistic regression. The binary model assessed associations between LASSI-L scores and the presence or absence of MCI/AD, with variables entered using a stepwise backward method. Three models were constructed: a full model including all nine variables, and a reduced model including four variables selected based on the stepwise approach using the likelihood-ratio test and changes in information criteria (AIC and BIC). The reduced model was adjusted for age (in years), years of education, and comorbidity (Charlson Comorbidity Index score), which were considered potential confounding variables for the outcome.

The ordinal model estimated the likelihood of more severe cognitive impairment. Initially, a proportional odds model was fitted using the Backward method with all nine variables, which identified the same four predictors as the binary model. These were then tested in proportional odds and generalized ordinal models. Since the proportional odds assumption was not met, the generalized model provided a better fit according to log-likelihood. Results for both models are reported as odds ratios (OR) with 95% confidence intervals, and regression assumptions were verified through residual analysis.

A *p*-value < 0.05 was considered statistically significant. All analyses were performed using SPSS (version 21), and the binary and ordinal logistic regression models were conducted in R (version 4.4.3).

## 3. Results

### 3.1. Descriptive Analysis

The results of the descriptive analysis are presented in Table 1. Statistically significant differences (*p* < 0.0001) were observed in almost all evaluated variables between groups. The CHG group had a mean age of 64.68 years, whereas the mild AD group averaged 78.44 years. A marked decrease in years of schooling was noted as the severity of cognitive impairment increased, with the CHG group averaging 16.34 years of education compared to 8.24 years in the AD group. The presence of comorbidities was similar across all three groups. Regarding global cognitive functioning, the CHG group scored an average of 26, the MCI group 18, and the mild AD group 15.

### 3.2. Discriminant Validity

To evaluate the discriminant validity of the instrument, an analysis of variance (ANOVA) was conducted, with results shown in Table 2. Mean scores and standard deviations for the different LASSI-L measures are presented across the three groups. This analysis revealed statistically significant differences (*p* < 0.0001) in all LASSI-L variables. Performance demonstrated a progressive decline across groups, with the mild AD group showing the lowest scores and greatest difficulty in free recall tasks (e.g., FRA1 means: 9.45 for CHG, 6.70 for MCI, 5.39 for mild AD) and cued recall (CRA2 means: 13.46 for CHG, 10.72 for MCI, 9.47 for mild AD). Cues improved recall less effectively in impaired individuals compared to cognitively healthy participants. Cued recall scores were higher than free recall scores for List B, suggesting a greater effect of proactive interference (PI) across groups.

### 3.3. Concurrent Validity

Regarding concurrent validity, correlation results are presented in Table 3. Statistically significant associations (*p* < 0.01) were found among the indices. Notably, the HVLT-H variables EIL1, EDL1, EDC1, EDL2, and EDC2 showed moderate to strong correlations with LASSI-L indices FRA1, CRA1, CRA2, FRB1, CRB1, and CRB2. EDL1 exhibited correlation coefficients above 0.45 across all associations, particularly with CRA2 (0.550) and CRB2 (0.552), indicating a robust relationship between the two instruments at delayed recall. EII1 showed lower but still significant correlations (ranging from 0.229 to 0.353), suggesting a moderate association with other dimensions.

### 3.4. Predictive Validity

For predictive validity, first, a binary logistic regression model was performed to estimate the probability of MCI or mild AD based on LASSI-L scores and to identify which scores were most strongly associated with diagnosis. A stepwise backward procedure was applied, starting with all scores and sequentially removing non-significant variables. The results are summarized in Table 4. Three models were generated: the initial full model, a reduced model including only significant predictors from the full model, and a final reduced and adjusted model controlled for age, education, and comorbidity. The full model showed an odds ratio (OR) of 0.736 (95% CI: 0.630–0.860, *p* = 0.000), while the reduced and adjusted model yielded an OR of 0.720 (95% CI: 0.616–0.843, *p* < 0.0001). In the adjusted model, each additional point in CRA2 significantly reduced the likelihood of cognitive impairment, even after controlling for age and education. FRB1 was the other score consistently associated with outcome across all models (OR = 0.745, 95% CI: 0.622–0.893, *p* = 0.001). Other scores (FRA1, CRA1, CRB1, SdCRA, DR) lost significance in the reduced and adjusted model, indicating their effects were confounded by sociodemographic factors. Age (OR = 1.114, 95% CI: 1.060–1.170, *p* < 0.0001) and education (OR = 0.835, 95% CI: 0.778–0.896, *p* < 0.0001) were inversely associated with cognitive impairment, whereas comorbidity (OR = 1.001; *p* = 0.382) showed no significant effect.

Second, a generalized ordinal logistic regression model, which did not assume proportional odds, was conducted to assess whether LASSI-L performance predicted transitions between cognitive states. Results (Table 5) indicated that CRA2 (OR = 0.73, *p* < 0.001) and FRB1 (OR = 0.75, *p* < 0.001) were significantly associated with lower odds of progression from normal cognition to MCI, suggesting a protective effect. However, these scores did not significantly predict progression from MCI to mild AD. Increasing age significantly influenced both transitions (OR ≥ 1.11, *p* < 0.001), while education primarily protected against the initial transition from CHG to MCI (OR = 0.87, *p* < 0.001). The effect of education on dementia progression approached but did not reach significance (*p* = 0.058). The Charlson comorbidity index was not associated with cognitive diagnosis.

## 4. Discussion

Current evidence suggests that certain memory tests—particularly those assessing free and cued recall—can detect the specific clinical phenotype associated with typical Alzheimer’s disease (AD) during its preclinical and mild stages, such as mild cognitive impairment (MCI). The value of these tests lies in enabling earlier and more timely diagnoses, especially in contexts like Latin America, where biomarker-based diagnosis is not widely accessible. However, most of these instruments have not yet been validated in Latin American populations, including Mexico. The LASSI-L exemplifies this type of tool, and thus the present study sought to validate the LASSI-L among cognitively healthy Mexican subjects, as well as individuals with MCI and mild AD.

To this end, analyses of discriminant, criterion, and predictive validity were conducted. Discriminant validity was demonstrated through analysis of variance and post hoc tests, which revealed significant differences across the three groups (CHG, MCI, and AD) in all LASSI-L indices, confirming the instrument’s discriminative capacity. As expected, scores declined progressively with increasing severity of cognitive impairment, consistent with literature showing that AD-related cognitive deficits become more severe and widespread as the disease progresses [43]. Importantly, individuals in the preclinical and early stages show deficits in using semantic cues to retrieve information, reflecting neurodegeneration in medial temporal regions such as the hippocampus [44]. Notably, recovery from proactive interference (CRB2) emerged as the measure with the largest group differences. This result aligns with prior evidence indicating that performance on this particular subtest has high discriminative properties, even among cognitively healthy individuals [29].

Criterion validity was supported by correlation analyses demonstrating significant associations between analogous indices from the LASSI-L and the Hopkins Verbal Learning Test (HVLT). High correlations were particularly evident between delayed recall with cues (EDL1, EDC2 on HVLT and CRA2, CRB2 on LASSI-L). Lower, though still significant, correlations likely reflect methodological differences: while HVLT follows a traditional serial word learning paradigm, LASSI-L incorporates controlled, in-depth learning paradigms. These findings underline the conceptual coherence between the instruments despite procedural differences [27].

Regarding predictive validity, logistic regression models revealed that LASSI-L performance is significantly associated with MCI and mild AD diagnoses, aligning with previous reports [26,27,28]. Notably, LASSI-L scores related to semantic cuing and proactive interference (CRA2 and FRB1) remained significant predictors even after adjusting for age, education, and comorbidity. This suggests that these LASSI-L indices may serve as markers relatively independent of education, a particularly valuable attribute in Mexico where educational attainment varies widely [45]. This is crucial given the predominance of low educational levels in the older Mexican population [46] and evidence that education interacts with age to influence cognitive performance [47]. Age itself is a well-known factor affecting cognitive test performance [48], especially memory, which is particularly vulnerable to aging [49]. The retention of the association after adjusting for age highlights the LASSI-L’s robustness against such biases, an important consideration given the heterogeneity of aging processes across Latin America [50].

Finally, findings from the ordinal regression model emphasize the role of cued recall (CRA2) and proactive interference (FRB1) as risk factors for transitioning from normal cognition to MCI. This highlights LASSI-L potential for differential diagnosis when MCI etiology is not clear, also this aligns with evidence that individuals with MCI are more susceptible to proactive interference due to deficits in information encoding [51], highlighting the importance of assessing these effects in populations at increased risk for AD progression. It also supports prior work indicating that while memory tests emphasizing cued recall are superior in detecting early AD-related changes, they may not be as effective in predicting conversion to dementia [9]. Additionally, years of schooling emerged as a protective factor against the transition from normal cognition to MCI but did not significantly influence progression from MCI to AD, consistent with the cognitive reserve hypothesis that education delays symptom onset primarily in earlier disease stages [52].

Several limitations should be noted. Despite comprehensive clinical evaluation and specialist diagnosis, the absence of biomarker data limits the ability to confirm prodromal AD etiology in most MCI cases, reflecting a common challenge in Mexico and Latin America where biomarker access is limited. The cross-sectional design also restricts conclusions regarding the LASSI-L’s capacity to predict conversion from MCI to AD, highlighting the need for longitudinal studies.

## 5. Conclusions

To our knowledge, the present study is the first one in Latin America to demonstrate that the Loewenstein–Acevedo Scales of Semantic Interference and Learning (LASSI-L) is a valid tool, capable of distinguishing cognitively healthy older Mexican adults from those with varying degrees of cognitive impairment. The test’s strength lies particularly in measures related to cued recall and proactive interference effects. Its utility in detecting memory disturbances in individuals with MCI represents an important advancement for the Latin American context, where biomarker-based diagnostics are scarce and validated neuropsychological tests for subtle cognitive changes are lacking. Validating the LASSI-L in the Mexican population represents a critical step toward more contextualized, accessible, and cost-effective neuropsychological assessment. This instrument can enhance early detection and diagnosis, inform timely intervention planning, and promote equitable access to quality cognitive evaluation, even in resource-limited settings.

## Figures and Tables

**Table 1:** Descriptive analysis.

	Total	CHG *n* = 156	MCI *n* = 127	AD *n* = 72	*p* Value
	Mean (SD)	Mean (SD)	Mean (SD)	Mean (SD)	
Age in years	69.77 (8.83)	64.68 (6.67)	71.07 (7.55)	78.44 (7.36)	0.152
Sex					
Men	68 (19.2)	116 (74.4)	107 (84.3)	63 (88.7)	0.006
Women	286 (80.8)	40 (25.6)	20 (15.7)	8 (11.3)	
Years of schooling	12.03 (6.62)	16.34 (5.59)	8.90 (5.12)	8.24 (5.59)	0.251
Occupation					
Employed (paid)	265 (74.6)	85 (54.5)	109 (85.8)	71 (98.6)	<0.0001
Unemployed/Not employed (no paid activity)	79 (22.3)	65 (41.7)	13 (10.2)	1 (1.4)	
Eventually employed	11 (3.1)	6 (3.8)	5 (3.9)	0 (0.0)	
Comorbidity *	3.1 (1.7)	2.5 (1.5)	3.2(1.7)	4.3(2)	0.438
Dependence **	97.37 (10.80)	100 (0)	100 (0)	87.39 (21.24)	<0.0001
Functionality ***	7.35 (1.60)	8.00 (0.00)	7.96 (0.23)	4.88 (2.21)	<0.0001
MOCA	21.15 (6.16)	26.26 (1.67)	18.28 (4.81)	15.11 (5.71)	<0.0001

* Charlson comorbidity index, ** Barthel’s basic activities of daily living, *** Lawton and Brody functional activities of daily living.

**Table 2:** Discriminant validity, analysis of variance (ANOVA).

	Total Mean (SD)	CHG Mean (SD) *n* = 156	MCI Mean (SD) *n* = 1 27	AD Mean (SD) *n* = 72	F	Partial η^2^	*p* Value
FRA1	7.64 (3.14)	9.45 (2.76)	6.70 (2.59) ^a^	5.39 (2.61) ^a,b^	69.2	0.289	<0.0001
CRA1	9.06 (2.96)	10.94 (2.34)	7.75 (2.56) ^a^	7.33 (2.52) ^a^	81.4	0.313	<0.0001
CRA2	11.67 (2.99)	13.46 (1.85)	10.72 (2.88) ^a^	9.47 (2.95) ^a,b^	77.3	0.318	<0.0001
FRB1	5.59 (2.70)	7.29 (2.25)	4.54 (2.30) ^a^	3.75 (2.01) ^a,b^	84.6	0.329	<0.0001
CRB1	6.48 (2.63)	8.01 (2.35)	5.59 (2.12) ^a^	4.75 (2.17) ^a,b^	67.9	0.273	<0.0001
CRB2	9.81 (3.10)	11.82 (2.32)	8.73 (2.59) ^a^	7.35 (2.65) ^a,b^	98.2	0.364	<0.0001
SdFRA	5.42 (3.26)	7.36 (2.92)	4.33 (2.77) ^a^	3.13 (2.24) ^a,b^	74.3	0.293	<0.0001
SdCRA	8.28 (2.98)	9.65 (2.74)	7.64 (2.71) ^a^	6.43 (2.56) ^a,b^	40.8	0.188	<0.0001
DR	22.29 (74.92)	20.06 (4.15)	14.90 (4.77) ^a^	11.25 (6.59) ^a,b^	85.9	0.334	<0.0001

Post hoc comparisons ^a^: significant differences (*p* < 0.05) with respect to the normal cognitive status group, ^b^: significant difference (*p* < 0.05) with respect to the moderate cognitive impairment group. Free recall List A (FRA1), Free recall List B (FRB1), Semantically cued recall List A1 (CRA1), Semantically cued recall List A2 (CRA2), Semantically cued recall List B1 (CRB1), Semantically cued recall List B2 (CRB2), Free deferred recall (SdFR), semantically cued deferred recall (SdCRA) and delayed total recall (DR).

**Table 3:** Concurrent validity, correlation analysis.

	FRA1	CRA1	CRA2	FRB1	CRB1	CRB2	SdFRA	SdCRA	DR
IFR1	0.465 **	0.499 **	0.515 **	0.470 **	0.398 **	0.563 **	0.441 **	0.492 **	0.549 **
IRI1	0.278 **	0.240 **	0.245 **	0.264 **	0.229 **	0.303 **	0.353 **	0.287 **	0.326 **
DLR1	0.469 **	0.541 **	0.550 **	0.455 **	0.348 **	0.552 **	0.501 **	0.513 **	0.498 **
DLRC1	0.423 **	0.473 **	0.494 **	0.436 **	0.427 **	0.516 **	0.483 **	0.505 **	0.543 **
DLR2	0.456 **	0.484 **	0.548 **	0.529 **	0.412 **	0.590 **	0.399 **	0.460 **	0.499 **
DLRC2	0.480 **	0.465 **	0.542 **	0.471 **	0.401 **	0.575 **	0.421 **	0.414 **	0.537 **

**. The correlation is significant at the 0.01 level (two-tailed).

**Table 4:** Predictive validity of LASSI-L scores and the presence of MCI/AD throughout binary logistic regression model.

	Full Model		Reduced Model		Reduced Adjusted Model	
	OR (95%CI)	*p* Value	OR (95%CI)	*p* Value	OR (95%CI)	*p* Value
FRA1	1.074 (0.899–1.284)	0.433	-		-	
CRA1	0.843 (0.692–1.026)	0.088	-		-	
CRA2	0.749 (0.640–0.876)	0.000	0.722 (0.624–0.837)	<0.0001	0.720 (0.616–0.843)	<0.0001
FRB1	0.797 (0.664–0.957)	0.015	0.749 (0.643–0.873)	<0.0001	0.745 (0.622–0.893)	0.001
CRB1	0.951 (0.799–1.131)	0.569	-		-	
CRB2	0.844 (0.716–0.995)	0.043	0.805 (0.694–0.932)	0.004	0.948 (0.794–1.131)	0.551
SdFRA	0.825 (0.708–0.962)	0.014	0.838 (0.744–0.943)	0.003	0.928 (0.805–1.070)	0.303
SdCRA	1.100 (0.936–1.291)	0.248	-		-	
DR	0.949 (0.870–1.035)	0.236	-		-	
Age	-		-		1.114 (1.060–1.170)	<0.0001
Years of schooling	-		-		0.835 (0.778–0.896)	<0.0001
Comorbidity	-		-		1.001 (0.999–1.003)	0.382

**Table 5:** Predictive validity, generalized ordinal logistic regression model.

	Mild Cognitive Impairment		Dementia	
	OR (95%CI)	*p* Value	OR (95%CI)	*p* Value
CRA2	0.734 (0.634–0.849)	<0.0001	0.888 (0.769–1.026)	0.106
FRB1	0.748 (0.633–0.883)	<0.0001	0.916 (0.772–1.086)	0.313
CRB2	0.965 (0.818–1.137)	0.667	0.902 (0.769–1.057)	0.211
SdFRA	0.926 (0.813–1.056)	0.252	0.885 (0.759–1.032)	0.103
Age	1.115 (1.066–1.167)	<0.0001	1.161 (1.104–1.221)	<0.0001
Years of schooling	0.866 (0.814–0.921)	<0.0001	0.943 (0.881–1.009)	0.058
Comorbidity	1.001 (0.998–1.003)	0.339	1.000 (0.997–1.002)	0.989

## Data Availability

Data is unavailable due to privacy and ethical restrictions.

## Abbreviations

The following abbreviations are used in this manuscript:

AD	Alzheimer’s disease
MCI	Mild Cognitive Impairment
FCSRT	Free and Cued Selective Reminding Test
LASSI-L	Loewenstein–Acevedo Scales of Semantic Interference and Learning
INGER	National Institute of Geriatrics
mND	Minor Neurocognitive Disorder
MND	Mayor Neurocognitive Disorder
CHG	cognitively healthy group
MoCA	Montreal Cognitive Assessment
HVLT	Hopkins Verbal Learning Test
CDR	Clinical Dementia Rating Scale
FRA1	Free recall List A
FRB1	Free recall List B
CRA1	Semantically cued recall List A1
CRA2	Semantically cued recall List A2
CRB1	Semantically cued recall List B1
CRB2	Semantically cued recall List B2
SdFR	Free deferred recall
SdCRA	Semantically cued deferred recall
DR	Total Delayed Recall
IFR1	Immediate recall free one
IRI1	Immediate recall interference one
DLR1	Delayed recall free one
DLRC1	Delayed recall with cues one
DLR2	Delayed recall free two
DLRC2	Delayed recall with cues two
